# The rising wave of cathinone use in people attending harm reduction facilities: a French repeated cross-sectional study (2019–2023)

**DOI:** 10.1186/s13722-026-00655-5

**Published:** 2026-03-26

**Authors:** Julia de Ternay, Anthony Plasse, Benjamin Rolland

**Affiliations:** 1 Service Universitaire d’Addictologie de Lyon (SUAL), Hospices Civils de Lyon, Lyon, France; 2 Research on Healthcare Performance (RESHAPE), INSERM U1290, Claude Bernard University Lyon 1, Lyon, France; 3 CAARUD Pause Diabolo, Le MAS, 17 rue Crépet, Lyon, 69007 France; 4 Service Universitaire d’Addictologie de Lyon (SUAL), Le Vinatier, Bron, France; 5 UMR 5292 CNRS, U1028 INSERM, Université Lyon 1, CRNL, Équipe PSYR2, Bron, France

**Keywords:** Cathinone, PWUD, Stimulant, Psychostimulant, Harm reduction center

## Abstract

**Background:**

In Europe, cocaine use has recently been on the rise among people who use drugs (PWUD). In parallel, cathinones, another type of psychostimulant that were once associated within the unique context of chemsex, are now spreading to broader patterns of use. Here, we examined trends in reported drug use, including cathinones, among PWUD attending the main harm reduction center in Lyon, France, between 2019 and 2023.

**Methods:**

We conducted a year-by-year comparison of standardized assessments completed at entry and updated annually for all PWUD attending the harm reduction center. These assessments collected sociodemographic characteristics (i.e., age, sex, nationality, family, housing, and occupational status), and the types of drugs reported as used. We performed logistic regression models to compare the prevalence of each substance, used as the dependent variable, with year and sociodemographic characteristics of PWUD as independent variables. Adjusted odds ratios and their 95% confidence intervals (aOR [95% CI]) were reported.

**Results:**

A total of 1,652 PWUD were analyzed. Between 2019 and 2023, the proportion of PWUD reporting cathinone use increased from 1.1% to 10.9%, and from 53.2% to 73.2% for cocaine (*p* < 0.001 for both). In parallel, the proportion of PWUD reporting alcohol use decreased from 29.5% to 12.8%, and from 72.1% to 39.9% for opioids (*p* < 0.001 for both). After adjusting for age, sex, and socioeconomic factors, and using 2019 as the reference year, we found significant increases in reported cathinone use in 2022 (aOR [95% CI] = 4.84 [1.35–31.05]) and 2023 (aOR [95% CI] = 4.90 [1.40-31.06]), along with significant decreases in opioid use in 2022 (aOR [95% CI] = 0.50 [0.28–0.88]) and 2023 (aOR [95%CI] = 0.39 [0.22–0.66]). The changes observed for alcohol and cocaine were no longer significant.

**Conclusions:**

We observed a significant increase in the proportion of PWUD reporting cathinone use at harm reduction centers. This trend highlights the need for improved training for caregivers and adaptations in the services provided for these substances within these facilities. Further research is needed to gain a deeper understanding of the profiles of more vulnerable cathinone users and their motivations for use.

**Supplementary Information:**

The online version contains supplementary material available at 10.1186/s13722-026-00655-5.

## Background

Over recent years, drug use patterns have changed considerably in both North America and Europe. The opioid crisis affecting the USA since the 1990s has been marked by the emergence of a new wave of molecules, primarily fentanyl and its analogs [1], along with a sharp increase in drug overdose deaths, which now exceed 100,000 annually [2]. On the other side of the Atlantic, cocaine and synthetic psychostimulants, such as amphetamine, methamphetamine, synthetic cathinones, and 3,4-methylenedioxymethamphetamine (MDMA) now play a preeminent role in Europe’s drug problems [3]. In both Europe and North America, polydrug use is also a rising concern, particularly when sedative drugs such as opioids are mixed with psychostimulants, as the associations between drugs and families of drugs induce even more harm [1, 3].

In line with many other European countries, France has seen a dramatic increase in the use of psychostimulants, in particular cocaine, for which the estimated rates of use in the last 12 months have moved from 1.1% of the adult population in 2014 to 2.7% in 2023, and MDMA, whose rates moved from 0.9% to 1.8% during the same period [4]. Relative to some other European countries, in particular Scandinavian countries, the use of amphetamine and methamphetamine has remained relatively limited in France [4]. According to the French national OPPIDUM study, which monitors substance use among people who use drugs (PWUD) attending addiction and harm reduction facilities, 30% report cocaine use in 2023 and a rise of new synthetic molecules including cathinones has been observed in the recent years [5]. Historically, cathinones were initially used in the unique context of chemsex in populations of men who have sex with men (MSM), but now tend to be used in much broader contexts, as other psychostimulants [6–9]. In France, only local and unquantified accounts have reported on this emerging pattern of cathinone use outside the context of chemsex, essentially in festive settings [10, 11]. However, no longitudinal data have supported these accounts, particularly in samples of PWUD attending harm reduction centers.

France has a national system of harm reduction facilities, which are dedicated to providing sterile material for drug use and/or naloxone kits, to offering social and psychological support and prevention programs, and to ensuring basic medical screening and referral to treatment for diseases related to drug use (e.g., viral hepatitis) [12]. These centers target the most precarious PWUD, a population of people who are likely to be more frequently isolated, unemployed, and in precarious housing situations [13]. Thus, exploring changes in drug use patterns in this specific population is important, as it provides data on the most vulnerable PWUD. Whereas national reports aim to provide epidemiological data on drug use rates in what tends to be a representative sample of the global population of PWUD, exploring the profile changes in PWUD seeking support in harm reduction facilities gives more information about the drugs that generate a request for assistance in the most vulnerable part of the PWUD population.

Here, we explored the evolution in the patterns of drugs reported to be used by PWUD attending the main harm reduction center of Lyon, France, from 2019 to 2023, to appraise whether this population displayed similar changes to those observed in national surveys.

## Methods

### Type of study and participants

This was a retrospective observational study describing the characteristics of all PWUD attending the main harm reduction center of the city. Participants were all adult PWUD seeking support at the harm reduction center between 2019 and 2023, which could include the acquisition of supplies for substance use, request for drug testing, access to social work services, medical support, or other harm reduction services. To be assisted in the harm reduction center, PWUD had to be aged 18 years or more.

### Data collected

Data were retrospectively reviewed using the electronic data records of the center. Data were collected during the first interview at entry and were also updated on an annual basis for all users still coming to the center. The types of data collected included the year of first visit (categorical variable), and the number of visits of the participants during the same year. In addition, the following sociodemographic characteristics were collected: age (in years), sex (male or female; “other” sex was not included), current active job (yes or no), and stable housing (yes or no).

Moreover, we collected the list of substances reported by PWUD. This list is systematically assessed at the first visit and updated annually for those who continue to attend the center. The list consisted of the following substances: (i) alcohol, (ii) opioids, e.g., heroin, tramadol, codeine, fentanyl, or morphine, (iii) cocaine (crack or powder form), (iv) cannabis, (v) cathinones, e.g., mephedrone, 4-methylethylcathinone (4-MEC), 3-methylmethcathinone (3-MMC), or methylenedioxypyrovalerone (MDPV), (vi) ketamine, (vii) synthetic cannabinoids, (viii) LSD or other serotoninergic psychedelics, (ix) amphetamines, e.g., amphetamine, methamphetamine, or MDMA; (x) benzodiazepines; and (xi) other.

For each substance, it was noted whether the PWUD was using it (yes or no). Thus, for each PWUD, the total number of categories of substances for which they were seeking support in the harm reduction center was noted as a quantitative variable.

### Statistical analyses

Descriptive statistics are presented as the number and percentage for categorical variables, and the mean plus standard deviation (m ± SD) and the median and interquartile range (med[IQR] for quantitative variables. Bivariable comparisons compared the subsamples of PWUD of each available year, and the rest of the variables collected. Comparisons of categorical variables were performed either using the chi-squared test or the Fisher’s exact test, while comparisons of quantitative variables were performed using the Kruskal-Wallis test.

In order to identify whether annual variations in drug use could be related to changes in the sociodemographic profile of PWUD, we built independent logistic regression models in which the report of the category of substance used was the dependent variable, while the year, and relevant sociodemographic characteristics were the independent variables. In accordance with statistical recommendations for building logistic regression models [11], in the bivariable comparisons, all variables with a p-value of 0.1 or less were selected for inclusion into the logistic regression models.

For each substance type, two models of logistic regression were built. The first model was adjusted for age and sex, while the second model included all significant sociodemographic characteristics as adjustment variables. Since missing data resulted from inconsistent data collection at the harm reduction center, these were considered as missing at random and imputed, using a random forest multiple imputation [14]. We chose this model of imputation as it is non-parametric and has a robust performance with high-dimensional and non-linear data structures, making it suitable for the present dataset that includes categorical variables. We used the {MissForest} package for random forest imputations [15]. As the quality of imputation tends to deteriorate with rates of missing data higher than 40%, variable with more than 40% missing data were not imputed. Thus, participants with remaining missing data after imputation were excluded from the logistic regressions.

The significance level of logistic regression models was defined as *p* < 0.05. All statistical analyses were performed using XLSTAT 2023 (https://www.xlstat.com/en/) and RStudio (version 2023-09-1 + 494).

## Results

### Descriptive statistics and bivariable analyses

In total, data from 1,652 PWUD were collected. The descriptive statistics and the results of the bivariable comparisons are displayed in Table 1. Overall, annual differences were found regarding age, nationality, and professionality (*p* < 0.01 for all tests). No difference was found regarding housing conditions. There was an increase in PWUD visiting the harm reduction center from 2019 (*n* = 190) to 2023 (*n* = 552). However, the average number of visits by PWUD progressively decreased between 2019 and 2023, and went from med = 13 IQR [3–33] in 2019, to med = 3, IQR [1–9] in 2023 (*p* < 0.001).

**Table 1:** Characteristics of PWUD seeking support in the harm reduction center (non-imputed dataset)

Variable		Missing	Total Sample (*n* = 1,652)	2019 (*n* = 190)	2020 (*n* = 188)	2021 (*n* = 295)	2022 (*n* = 427)	2023 (*n* = 552)	*p*-value
Age	*m ± SD; med [IQR]*	266	42.0 ± 10.1; 43 [32–49]	43.5 ± 9.7; 44 [46–50]	44.3 ± 9.1; 45 [37–49]	42.1 ± 10.0; 43 [36–48]	41.3 ± 10.4; 42 [35–49]	41.1 ± 10.4; 43 [34–48]	**
Female Gender	*n (%)*	0	312 (18.9%)	37 (19.5%)	40 (21.3%)	59 (20.0%)	77 (18.0%)	99 (17.9%)	NS
French Nationality	*n (%)*	541	846 (76.1%)	106 (67.5%)	103 (69.6%)	136 (72.7%)	221 (79.2%)	280 (82.4%)	**
Stable Housing	*n (%)*	511	504 (44.1%)	60 (41.7%)	57 (41.9%)	65 (36.9%)	132 (47.1%)	190 (46.9%)	NS
Professionally active	*n (%)*	758	266 (29.7%)	17 (18.7%)	24 (22.2%)	38 (26.8%)	80 (36.7%)	107 (31.9%)	**
Yearly number of visits	*m ± SD; med [IQR]*	109	13.1 ± 24.3; 4 [1–13]	24.7 ± 29.6; 13 [3–33]	23.4 ± 37.2; 8 [3–28]	14.4 ± 28.1; 5 [2–13]	9.1 ± 18.3; 3 [1–9]	8.1 ± 14.5; 3 [1–9]	***
Total number of substances	*m ± SD; med [IQR]*	0	1.7 ± 0.9; 1 [1–2]	1.8 ± 1.0; 2 [1–2]	1.9 ± 1.0; 2 [1–2]	1.7 ± 1.0; 1 [1–2]	1.7 ± 0.9; 1 [1–2]	1.7 ± 1.0; 1 [1–2]	**
Requiring support for:									
Alcohol	*n (%)*	0	253 (15.3%)	56 (29.5%)	38 (20.2%)	39 (13.2%)	49 (11.5%)	71 (12.8%)	***
Opioids	*n (%)*	0	815 (49.3%)	137 (72.1%)	118 (62.8%)	151 (51.2%)	189 (44.2%)	220 (39.9%)	***
Cocaine	*n (%)*	0	1,101 (66.6%)	101 (53.2%)	128 (68.1%)	182 (61.7%)	286 (67.0%)	404 (73.2%)	***
Cannabis	*n (%)*	0	241 (14.6%)	26 (13.7%)	29 (15.4%)	44 (14.9%)	59 (13.8%)	83 (15.0%)	NS
Cathinones	*n (%)*	0	127 (7.7%)	2 (1.1%)	5 (2.7%)	15 (5.1%)	45 (10.5%)	60 (10.9%)	***
Ketamine	*n (%)*	0	63 (3.8%)	3 (1.6%)	4 (2.1%)	8 (2.7%)	22 (5.1%)	26 (4.7%)	†
Synthetic cannabinoids	*n (%)*	0	60 (3.6%)	2 (1.1%)	6 (3.2%)	19 (6.4%)	17 (4.0%)	16 (2.9%)	*
LSD or other psychedelics	*n (%)*	0	44 (2.7%)	3 (1.6%)	5 (2.7%)	6 (2.0%)	11 (2.6%)	19 (3.4%)	NS
Amphetamines	*n (%)*	0	41 (2.5%)	4 (2.1%)	5 (2.7%)	6 (2.0%)	15 (3.5%)	11 (2.0%)	NS
Benzodiazepines	*n (%)*	0	35 (2.1%)	5 (2.6%)	3 (1.6%)	7 (2.4%)	8 (1.9%)	12 (2.2%)	NS
Other	*n (%)*	0	217 (13.1%)	17 (8.9%)	21 (11.2%)	45 (15.3%)	61 (14.3%)	73 (13.2%)	NS

* = p-value ≤ 0.05, ** = p-value < 0.01, *** = p-value < 0.001, † = p-value between 0.05 and 0.1

**Fig. 1:**
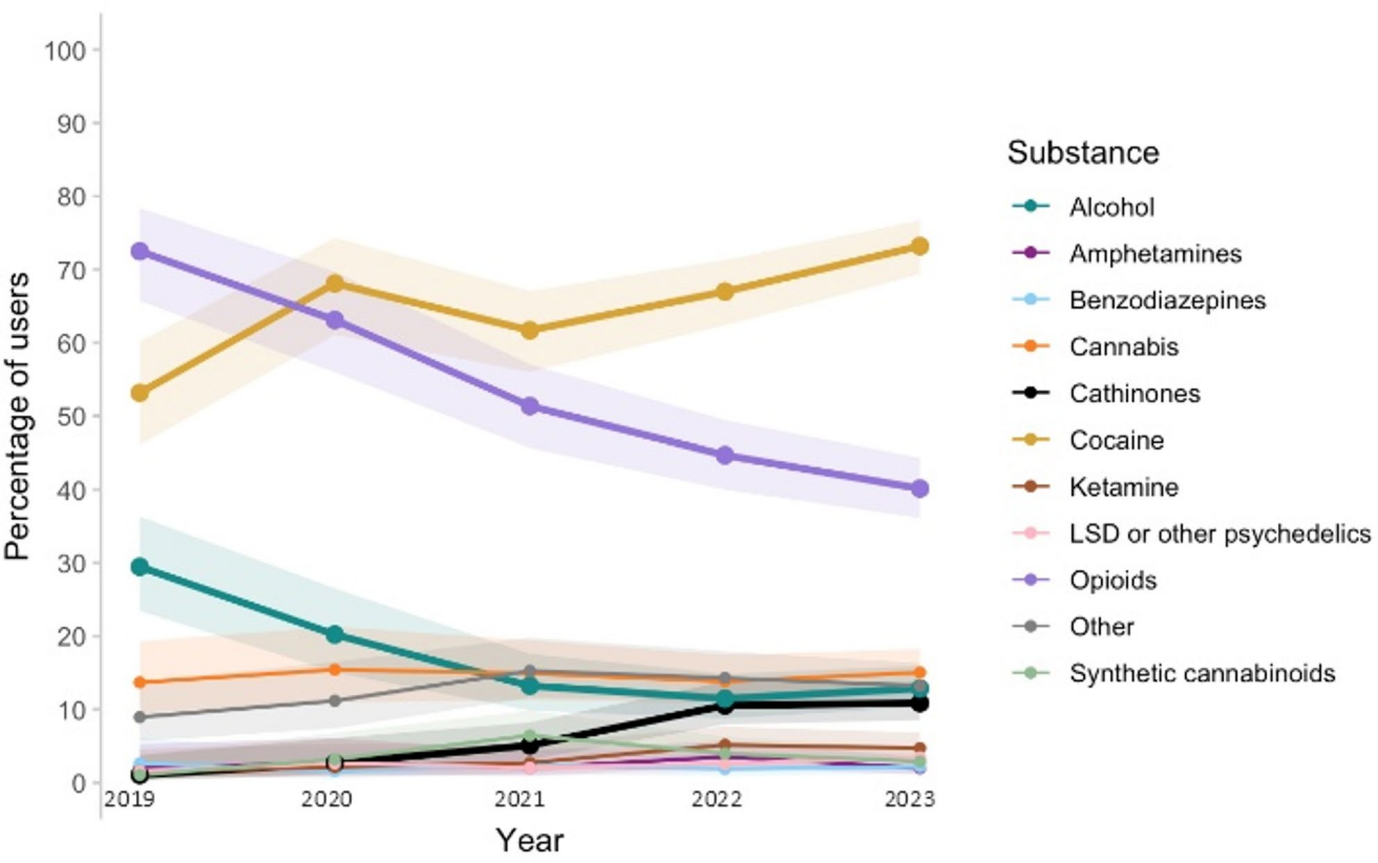
Trends in reported use of alcohol, opioids, cocaine, cathinones, and other substances among PWUD attending the harm reduction center, Lyon, 2019–2023

Trends in reported use of alcohol, opioids, cocaine, cathinones, and other substances among PWUD attending the harm reduction center, from 2019 to 2023, can be visualized in Fig. 1. There was a significant difference in the annual rates of PWUD reporting cocaine use, with annual rates increasing from 53.2% (*n* = 101) in 2019 to 73.2% (*n* = 404) in 2023 (*p* < 0.001). Furthermore, cathinone use was reported by 1.1% (*n* = 2) of PWUD in 2019, vs. 3.2% (*n* = 6) in 2020, 5.1% (*n* = 15) in 2021, 10.5% (*n* = 45) in 2022, and 10.9% (*n* = 60) in 2023, respectively (*p* < 0.001).

By contrast, the rates of reported opioid and alcohol use almost constantly decreased between 2019 and 2023. Alcohol use was reported by 29.5% (*n* = 56) of PWUD in 2019, vs. 20.2% (*n* = 38) in 2020, 13.2% (*n* = 39) in 2021, 11.5% (*n* = 49) in 2022, and 12.8% (*n* = 71) in 2023, respectively (*p* < 0.001). Similarly, opioid use was reported by 72.1% (*n* = 137) of PWUD in 2019, vs. 62.8% (*n* = 118) in 2020, 51.8% (*n* = 151) in 2021, 44.2% (*n* = 189) in 2022, and 39.9% (*n* = 220) in 2023, respectively (*p* < 0.001).

In addition, there was a significant but nonlinear difference in the yearly rates of reports of synthetic cannabinoid use, which peaked in 2021 with 6.4% (*n* = 19) of PWUD, and decreased to 2.9% (*n* = 16) of PWUD in 2023. There was also a trend toward an association between the year and ketamine use, although it was not statistically significant.

### Logistic regression models

When adjusted for age and sex (Model 1, *n* = 1,652), the analyses revealed that, relative to 2019, there was a significant increase in the odds of PWUDS reporting cocaine use in 2020, 2021, and 2023 (Table 2). Similarly, we found that PWUD had consistent increased odds of reporting cathinone use, relative to 2019 (aOR [95%CI] of 5.06 [1.40-32.37] in 2021, 10.97 [3.33–67.78] in 2022, and 11.32 [3.48–69.59] in 2023).

**Table 2:** Results of the logistic regression models analyzing the yearly effect of the likelihood of seeking support for alcohol, opioids, cocaine, and Cathinones (2019 as the reference), on the imputed dataset

Variable		2020 (aOR[95%CI]^1^)	2021 (aOR[95%CI])	2022 (aOR[95%CI])	2023 (aOR[95%CI])
**Model 1** *(n = 1*,*652)*					
Alcohol		**0.60 [0.37–0.96]** ^*^	**0.38 [0.24–0.60]** ^***^	**0.32 [0.21–0.50]** ^***^	**0.37 [0.25–0.56]** ^***^
Opioids		**0.63 [0.41–0.98]** ^*^	**0.41 [0.28–0.61]** ^***^	**0.33 [0.22–0.47]** ^***^	**0.27 [0.19–0.39]** ^***^
Cocaine		**1.90 [1.25–2.90]** ^**^	1.40 [0.96–2.03] ^†^	**1.76 [1.23–2.51]** ^**^	**2.37 [1.68–3.35]** ^***^
Cathinones		2.65 [0.56–18.69]	**5.06 [1.40-32.37]** ^*^	**10.97 [3.33–67.78]** ^**^	**11.32 [3.48–69.59]** ^***^
**Model 2** *(n = 894)*					
Alcohol		0.82 [0.41–1.61]	0.79 [0.41–1.52]	0.68 [0.37–1.28]	0.76 [0.43–1.37]
Opioids		0.80 [0.41–1.54]	0.76 [0.41–1.41]	**0.50 [0.28–0.88]** ^*^	**0.39 [0.22–0.66]** ^***^
Cocaine		1.33 [0.72–2.43]	1.08 [0.61–1.88]	1.17 [0.68–1.98]	1.65 [0.99–2.74] ^†^
Cathinones		1.95 [0.39–14.25]	2.18 [0.50-15.06]	**4.84 [1.35–31.05]** ^*^	**4.90 [1.40-31.06]** ^*^

^1^aOR [95%CI] = adjusted odd ratio [95% confidence interval], * = p-value ≤ 0.05, ** = p-value < 0.01, *** = p-value < 0.001, † = p-value between 0.05 and 0.1

In parallel, there was a significant decrease in the odds of reported alcohol use, which seemed to plateau from 2021 to 2023, with aORs [95%CI] remaining between 0.32 and 0.38, compared to 2019. By contrast, the odds of reported opioid use constantly declined, with an aOR [95%CI] of 0.63 [0.41–0.98] in 2020, 0.41 [0.28–0.61] in 2021, 0.33 [0.22–0.47] in 2022, and 0.27 [0.19–0.39] in 2023.

When adjusted for age, sex, French nationality, and professional status (Model 2, *n* = 894), significant differences from 2019 were still found for cathinones in 2022 (aOR [95%CI] = 4.84 [1.35–31.05]) and 2023 (aOR [95%CI] = 4.90 [1.40-31.06]), along with significant decreases in the odds of reported opioid use in 2022 (aOR[95%CI] = 0.50 [0.28–0.88]) and 2023 (aOR[95%CI] = 0.39 [0.22–0.66]). Results were no longer significant for other substances and other years.

Results with the non-imputed dataset can be found in the supplemental material, as well as a table containing the descriptive characteristics of PWUD reporting cathinone use.

## Discussion

The primary objective of this study was to examine the longitudinal changes in the types of substances for which PWUD sought support at Lyon’s main harm reduction center between 2019 and 2023. There was a marked increase in first-time visits to the harm reduction center during the study period, which could be explained by an intensified outreach strategy implemented since 2021 and by additional human resources allocated to the center. These efforts likely improved the center’s visibility and accessibility and therefore may have contributed to the rise in new attendances observed. However, in parallel, there was a decrease in the annual number of visits per PWUD. This pattern may suggest that, while more PWUD accessed harm reduction services over time, a growing proportion did so on a less regular or more occasional basis. Although no firm explanation can be drawn from our data, one hypothesis is that the strengthened outreach activities may have attracted a larger number of episodic visitors, while the relative proportion of long-term, frequent attendees declined.

Overall, we found that crude rates of reported cocaine and cathinone use increased, while rates for alcohol and opioids consistently declined over time. However, after adjusting for socio-economic factors, only cathinones showed a statistically significant increase, whereas opioids exhibited a significant decrease. The overall prevalence of cathinone users among PWUD visiting the harm reduction center in our sample appears higher than the 3.4% rate found in a 2020 French study focusing on a similar population [16]. While this could potentially reflect regional differences, yearly rates are similar in both studies. The rise in cathinone users seeking support at the harm reduction center in our study aligns with a documented increase in cathinone use in Paris and other European cities from 2019 to 2022, even outside the context of chemsex [9]. Our data also match recent findings, indicating that cathinones have become one of the most frequently detected substances in used syringes collected from automatic injection kit dispensers, public spaces, and harm reduction centers [9, 17, 18]. For instance, cathinones were detected in 89% of used syringes collected in Paris [9]. To the best of our knowledge, our study is the first to highlight this increase among PWUD attending a harm reduction center.

Several factors could account for this increase. Cathinones are now widely available across Europe [19]. Furthermore, they can be easily purchased online at a relatively low price [20], potentially making them a more affordable option for economically vulnerable users compared to other stimulants, especially cocaine. This affordability may also partly explain why, in contrast with general population trends, we found no significant rise in cocaine support-seeking after adjusting for professional status and French nationality in sensitivity analyses. Regional data in Lyon showed that cathinones were less expensive than cocaine when bought at street prices in 2023 [11]. However, price alone may not account for the trend, as we observed no change in rates of PWUD seeking support for other affordable stimulants, such as amphetamines [11]. A key implication of our findings is the urgent need to better understand cathinone use within this population. Future studies should specifically investigate factors unique to these substances, including their pharmacological effects, perceived harms, motivations for use in comparison with other stimulants, usage contexts, and addiction risks. In particular, it would be valuable to explore how users themselves distinguish cathinones from other stimulants, as this may shed light on the reasons behind the increasing trend observed in our study.

Another notable finding of our study is that the rates of PWUD reporting opioid use have regularly and significantly decreased in our sample from 2019 to 2023, despite a concurrent increase in support-seeking for opioid use at the national level during the same period [21]. While these results may reflect a broader shift in drug use patterns among local PWUD attending harm reduction facilities, an alternative explanation could involve changes in the profiles of PWUD using these services. Historically, cathinones are often used in the context of chemsex among MSM. Although we lack specific data on the context of cathinone use among PWUD in our sample, the rise in reported use of cathinones remained significant even after adjusting for sex, which may suggest that the trend observed is not solely due to an increase in chemsex-related cases. Interestingly, the characteristics of cathinone use among PWUD in our sample (see supplemental material) appear to differ from those of typical harm reduction center users [13, 16, 22]. This subgroup exhibits similarities with the described profile of recreational festive users [6–8], tending to be male, employed or students, and generally less economically vulnerable. This trend could indicate that a new population is now turning to harm reduction centers.

Whether the observed increase in PWUD reporting cathinone use in harm reduction centers reflects greater use among vulnerable PWUD or marks the arrival of new profiles within these centers, it raises important questions about how information and care services should be adapted. Cathinone use is associated with serious health risks including fatal intoxication, violence, and suicidal behavior [23–25], which both users and caregivers need to be aware of to mitigate these risks. Additionally, the diverse contexts of cathinone use (e.g. solitary use, use with other substances, recreational settings, chemsex) should be discussed with users to tailor harm reduction strategies. Specific measures could include access to clean supplies, information on adverse effects when combined with other substances, sexual health screening, and facilitated access to addiction-specific care.

We acknowledge several limitations in our study. First, since this was a single-center study, caution should be exercised when generalizing our findings to the broader population of PWUD attending harm reduction centers. Nonetheless, our sample appears to reflect similar substance use and socioeconomic characteristics reported in comparable populations. Second, given the relatively small proportion of cathinone users within our sample, in particular at the beginning of the study period, our statistical power was limited, resulting in wider confidence intervals for the rates of PWUD reporting cathinone use. Third, we lacked context regarding the use of cathinones, which means that we could only rely on assumptions about the reasons behind the increasing trend. Finally, substance use information was self-reported and may have been affected by biases such as recall bias and social desirability bias. Alternative approaches, such as wastewater analysis [26], have been used in other studies to monitor changes in cathinone use. While these methods also have their own limitations, participants in our study were informed that data collection was anonymous, which may have helped to mitigate some reporting bias.

In conclusion, we observed a significant increase in the rates of PWUD reporting cathinone use and seeking support at harm reduction centers. This trend highlights the need for improved training for caregivers and adaptations in the services provided for these substances within these facilities. Further research should be conducted to gain a deeper understanding of the profiles of more vulnerable cathinone users and their motivations for use.

## Supplementary Information

Below is the link to the electronic supplementary material.

Supplementary Material 1

## Data Availability

Datasets used and analyzed during the current study are available from the corresponding author on reasonable request.
