# Supplementary material for: The rising wave of cathinone use in people attending harm reduction facilities: a French repeated cross-sectional study (2019–2023)
Source: Addict Sci Clin Pract. 2026 Mar 26;21:31. doi: 10.1186/s13722-026-00655-5 (PMC13020218; doi:10.1186/s13722-026-00655-5)
Supplement: Supplementary file 1 — Supplementary Material 1 [file 13722_2026_655_MOESM1_ESM.docx]

**Supplemental material**

| Characteristic | 2019, N = 190 | 2020, N = 188 | 2021, N = 295 | 2022, N = 427 | 2023, N = 552 | Overall, N = 1,652 |
| --- | --- | --- | --- | --- | --- | --- |
| **Age** | 43.5 (9.5) 44.0 | 44.1 (8.6) 45.0 | 42.0 (9.1) 42.8 | 41.2 (9.4) 41.1 | 41.0 (9.7) 40.8 | 41.9 (9.4) 42.0 |
| **Female gender** | 37 (19.5%) | 40 (21.3%) | 59 (20.0%) | 78 (18.3%) | 101 (18.3%) | 315 (19.1%) |
| **French nationality** | 126 (66.3%) | 133 (70.7%) | 218 (73.9%) | 337 (78.9%) | 452 (81.9%) | 1,266 (76.6%) |
| **Stable housing** | 68 (35.8%) | 73 (38.8%) | 109 (36.9%) | 179 (41.9%) | 224 (40.6%) | 653 (39.5%) |
| **Professionally active** | 17 (18.7%) | 24 (22.2%) | 38 (26.8%) | 80 (36.7%) | 107 (31.9%) | 266 (29.8%) |
| *Unknown* | 99 | 80 | 153 | 209 | 217 | 758 |
| **Yearly number of visits** | 24.3 (29.0) 13.6 | 23.1 (36.2) 8.0 | 13.4 (26.6) 5.0 | 9.1 (17.8) 3.0 | 8.1 (14.1) 3.0 | 12.9 (23.6) 4.0 |
| **Total number of substances** | 1.8 (0.9) 2.0 | 1.9 (1.0) 2.0 | 1.7 (1.0) 1.0 | 1.7 (0.9) 1.0 | 1.7 (0.9) 1.0 | 1.7 (0.9) 1.0 |
| **TotalJ** | 1.9 (0.9) 2.0 | 1.9 (1.0) 2.0 | 1.8 (1.0) 2.0 | 1.8 (1.1) 1.0 | 1.8 (1.0) 2.0 | 1.8 (1.0) 2.0 |
| **Alcohol** | 56 (29.5%) | 38 (20.2%) | 39 (13.2%) | 49 (11.5%) | 71 (12.9%) | 253 (15.3%) |
| **Opioids** | 138 (72.6%) | 119 (63.3%) | 152 (51.5%) | 193 (45.2%) | 224 (40.6%) | 826 (50.0%) |
| **Cocaine** | 101 (53.2%) | 128 (68.1%) | 182 (61.7%) | 286 (67.0%) | 404 (73.2%) | 1,101 (66.6%) |
| **Cannabis** | 26 (13.7%) | 29 (15.4%) | 44 (14.9%) | 59 (13.8%) | 83 (15.0%) | 241 (14.6%) |
| **Cathinones** | 2 (1.1%) | 5 (2.7%) | 15 (5.1%) | 45 (10.5%) | 60 (10.9%) | 127 (7.7%) |
| **Ketamine** | 3 (1.6%) | 4 (2.1%) | 8 (2.7%) | 22 (5.2%) | 26 (4.7%) | 63 (3.8%) |
| **Synthetic cannabinoids** | 2 (1.1%) | 6 (3.2%) | 19 (6.4%) | 17 (4.0%) | 16 (2.9%) | 60 (3.6%) |
| **LSD** | 3 (1.6%) | 5 (2.7%) | 6 (2.0%) | 11 (2.6%) | 19 (3.4%) | 44 (2.7%) |
| **Amphetamines** | 4 (2.1%) | 5 (2.7%) | 6 (2.0%) | 15 (3.5%) | 11 (2.0%) | 41 (2.5%) |
| **Benzodiazepines** | 5 (2.6%) | 3 (1.6%) | 7 (2.4%) | 8 (1.9%) | 12 (2.2%) | 35 (2.1%) |
| **Other** | 17 (8.9%) | 21 (11.2%) | 45 (15.3%) | 61 (14.3%) | 73 (13.2%) | 217 (13.1%) |
| ^1^ Mean (SD) Median; n (%) |  |  |  |  |  |  |
|  |  |  |  |  |  |  |

**Table 1: characteristics of PWUD seeking support in the harm reduction center, after multiple imputation**

| Characteristic | 2019, N = 2 | 2020, N = 5 | 2021, N = 15 | 2022, N = 45 | 2023, N = 60 | Overall, N = 127 |
| --- | --- | --- | --- | --- | --- | --- |
| **AGE** | 42.5 (13.4) 42.5 | 40.2 (11.3) 37.0 | 38.4 (9.3) 37.0 | 41.9 (10.6) 39.0 | 41.3 (11.0) 41.0 | 41.2 (10.6) 38.0 |
| *Unknown* | 0 | 0 | 2 | 3 | 4 | 9 |
| **FEMALE GENDER** | 0 (0.0%) | 0 (0.0%) | 0 (0.0%) | 0 (0.0%) | 3 (5.1%) | 3 (2.4%) |
| *Unknown* | 0 | 0 | 0 | 0 | 1 | 1 |
| **FRENCH NATIONALITY** | 2 (100.0%) | 4 (100.0%) | 9 (100.0%) | 32 (100.0%) | 42 (97.7%) | 89 (98.9%) |
| *Unknown* | 0 | 1 | 6 | 13 | 17 | 37 |
| **STABLE HOUSING** | 2 (100.0%) | 4 (100.0%) | 9 (100.0%) | 32 (88.9%) | 45 (90.0%) | 92 (91.1%) |
| *Unknown* | 0 | 1 | 6 | 9 | 10 | 26 |
| **PROFESSIONALLY ACTIVE** | 1 (50.0%) | 3 (60.0%) | 3 (37.5%) | 21 (65.6%) | 31 (63.3%) | 59 (61.5%) |
| *Unknown* | 0 | 0 | 7 | 13 | 11 | 31 |
| **YEARLY NUMBER OF VISITS** | 1.0 (NA) 1.0 | 5.8 (5.6) 3.5 | 3.1 (3.0) 2.0 | 3.7 (3.6) 2.0 | 4.4 (4.4) 2.0 | 4.0 (4.0) 2.0 |
| *Unknown* | 1 | 1 | 1 | 3 | 3 | 9 |
| **TOTAL NUMBER OF SUBSTANCES** | 2.0 (1.4) 2.0 | 1.6 (0.9) 1.0 | 1.5 (0.6) 1.0 | 1.4 (0.7) 1.0 | 1.4 (0.7) 1.0 | 1.4 (0.7) 1.0 |
| ALCOHOL | 0 (0.0%) | 0 (0.0%) | 0 (0.0%) | 1 (2.2%) | 1 (1.7%) | 2 (1.6%) |
| **OPIOID** | 0 (0.0%) | 0 (0.0%) | 0 (0.0%) | 2 (4.4%) | 3 (5.0%) | 5 (3.9%) |
| **COCAINE** | 2 (100.0%) | 2 (40.0%) | 2 (13.3%) | 8 (17.8%) | 15 (25.0%) | 29 (22.8%) |
| **CANNABIS** | 0 (0.0%) | 1 (20.0%) | 2 (13.3%) | 3 (6.7%) | 5 (8.3%) | 11 (8.7%) |
| **KETAMINE** | 1 (50.0%) | 1 (20.0%) | 1 (6.7%) | 4 (8.9%) | 6 (10.0%) | 13 (10.2%) |
| **NPS** | 0 (0.0%) | 0 (0.0%) | 1 (6.7%) | 1 (2.2%) | 1 (1.7%) | 3 (2.4%) |
| **LSD** | 1 (50.0%) | 1 (20.0%) | 1 (6.7%) | 1 (2.2%) | 2 (3.3%) | 6 (4.7%) |
| **AMPHETAMINE** | 0 (0.0%) | 0 (0.0%) | 0 (0.0%) | 0 (0.0%) | 0 (0.0%) | 0 (0.0%) |
| **BENZODIAZEPINES** | 0 (0.0%) | 0 (0.0%) | 0 (0.0%) | 0 (0.0%) | 1 (1.7%) | 1 (0.8%) |
| **OTHER** | 1 (50.0%) | 1 (20.0%) | 4 (26.7%) | 11 (24.4%) | 14 (23.3%) | 31 (24.4%) |
| ^1^ Mean (SD) Median; n (%) |  |  |  |  |  |  |
|  |  |  |  |  |  |  |

**Table 2: characteristics of PWUD reporting cathinones use**

| **Variable** |  | **2020** *(aOR[95%CI])* | **2021** *(aOR[95%CI])* | **2022** *(aOR[95%CI])* | **2023** *(aOR[95%CI])* |
| --- | --- | --- | --- | --- | --- |
|  |  |  |  |  |  |
|  |  |  |  |  |  |
| **Model 1** *(n=1,379)* |  |  |  |  |  |
| Alcohol |  | 0.63 [0.38-1.04] ^†^ | **0.40 [0.24-0.64] ^**^** | **0.35 [0.22-0.55] ^***^** | **0.39 [0.26-0.59] ^***^** |
| Opioids |  | 0.65 [0.41-1.04] ^†^ | **0.49 [0.32-0.75] ^**^** | **0.34 [0.23-0.51] ^***^** | **0.29 [0.20-0.43] ^***^** |
| Cocaine |  | **1.97 [1.26-3.07] ^**^** | 1.45 [0.98-2.15] ^†^ | **1.73 [1.19-2.51] ^**^** | **2.36 [1.65-3.37] ^***^** |
| Cathinones |  | 2.89 [0.55-15.13] | **5.12 [1.14-23.03] ^*^** | **12.47 [2.97-52.30] ^**^** | **11.64 [2.80-48.38] ^**^** |
|  |  |  |  |  |  |
| **Model 2** *(n=719)* |  |  |  |  |  |
| Alcohol |  | 0.88 [0.43-1.78] | 0.79 [0.40-1.58] | 0.74 [0.38-1.43] | 0.80 [0.44-1.48] |
| Opioids |  | 0.81 [0.41-1.62] | 0.87 [0.45-1.68] | **0.53 [0.29-0.97] ^*^** | **0.44 [0.25-0.78] ^**^** |
| Cocaine |  | 1.39 [0.74-2.61] | 1.18 [0.65-2.15] | 1.25 [0.72-2.19] | 1.64 [0.97-2.80] ^†^ |
| Cathinones |  | 1.71 [0.29-10.13] | 1.47 [0.26-8.19] | **4.69 [1.13-21.35] ^*^** | **4.37 [1.03-19.47] ^*^** |
|  |  |  |  |  |  |

**Table 3: Results of the logistic regression models analyzing the yearly effect on the likelihood of seeking support for alcohol, opioids, cocaine, and cathinones (2019 as the reference), on the non-imputed dataset**

^1^aOR [95%CI]= adjusted odd ratio [95% confidence interval]
